# Unexplained worsening of parkinsonian symptoms in a patient with advanced Parkinson’s disease as the sole initial presentation of COVID-19 infection: a case report

**DOI:** 10.1186/s41983-021-00314-3

**Published:** 2021-05-17

**Authors:** Walaa A. Kamel, Ismail Ibrahim Ismail, Mohamed Ibrahim, Jasem Y. Al-Hashel

**Affiliations:** 1 Department of Neurology, Ibn Sina Hospital, Gamal Abdel Nasser Street, Sabah Medical Area, Sabah, Kuwait; 2 Department of Neurology, Beni-Suef University, Beni Suef, Egypt; 3 Department of Medicine, Faculty of Medicine, Health Sciences Centre, Kuwait University, Kuwait City, Kuwait

**Keywords:** COVID-19, SARS-CoV-2, Parkinson’s disease, Parkinsonism

## Abstract

**Background:**

Parkinson’s disease (PD) is a neurodegenerative condition that has been reported following viral infections in rare occasions. Several neurological complications have emerged in association with coronavirus disease 2019 (COVID-19), since its declaration as a pandemic. Herein, we present a novel case of unexplained worsening of PD as the sole initial presentation of COVID-19, in the absence of fever or respiratory symptoms.

**Case presentation:**

A 56-year-old male with advanced PD presented with severe rigidity, dystonic posturing of both feet, and confusion of 4 days duration. His condition progressed to an akinetic-rigid state and confusion during the following week, and a routine nasopharyngeal swab tested positive for severe acute respiratory syndrome coronavirus 2 (SARS-CoV-2) on the 9th day of onset. He developed fever and dyspnea later and was intubated on the 10th day.

**Conclusion:**

To our knowledge, worsening of PD symptoms as the sole initial manifestation of SARS-CoV-2 infection, in the absence of other cardinal features of COVID-19, has not been reported in the literature. We suggest testing for COVID-19 infection in patients with PD, especially advanced cases, who present with unexplained worsening of symptoms, even in the absence of COVID-19 cardinal features.

## Background

Since the emergence of severe acute respiratory syndrome coronavirus 2 (SARS-CoV-2) in Wuhan, China, in December 2019, and the declaration of coronavirus disease (COVID-19) as a pandemic on March 11, 2020, several neurological manifestations have been associated with this disease [1].

Parkinson’s disease (PD) is a neurodegenerative condition that has been reported in rare occasions following viral infections. Historically, the influenza epidemic of 1918 was associated with post-encephalitic parkinsonism; however, several other viruses were also reported including Epstein-Barr virus, varicella zoster, HIV, hepatitis C virus, West Nile virus, dengue virus, cytomegalovirus, and Japanese encephalitis virus [2].

The full impact of COVID-19 on PD is still not well established; however, several published case reports made the link between PD development and COVID-19 infection particularly intriguing. Herein, we present a novel case of unexplained severe rigidity, akinesia, bilateral foot dystonia, and confusion, as the sole initial manifestations of SARS-CoV-2 infection in a previously well-controlled patient with advanced PD, in the absence of other cardinal features of COVID-19.

## Case presentation

A 56-year-old male with advanced PD developed severe rigidity, dystonic posturing of both feet, and confusion of 4 days duration in November 2020. He is not known to have any chronic medical illness and has been diagnosed with PD 7 years ago, with initial left-sided tremors, bradykinesia, and rigidity. He had a strong positive family history of PD, but genetic testing was not done. His initial MRI of the brain and FDG-PET scan were normal, and he had a stable course on levodopa. He started to develop wearing-off and dyskinesia after few years which were manageable with drug adjustment.

Dopamine agonists (DA) were added, but the patient developed behavioral changes and psychotic features in the form of hallucination and delusions, which improved with the cessation of DA and small doses of quetiapine. Subsequently, with disease progression, levodopa-carbidopa intestinal gel (LCIG) was started with excellent improvement of wearing off symptoms and peak dose dyskinesia. After 2 years, and due to financial issues, the patient stopped LCIG and continued on levodopa with a total equivalent daily dose of 1497 mg and quetiapine 100 mg daily. His last clinical examination was in January 2020 with a stable clinical condition. His Hoehn and Yahr Scale (H&Y) score was 3, MDS-Unified Parkinson’s Disease Rating Scale (UPDRS) part III was 57/132 in off stage, Non-Motor Symptoms Scale (NMSS) was 233/360, and Montreal Cognitive Assessment (MoCA) score was 24/30. During the lockdown period amid the COVID-19 pandemic, he was compliant to treatment, maintaining his daily activities and communicating with the treating physician through telemedicine, and no drug adjustment was needed during this time.

He started to have severe rigidity and bradykinesia on the 1st of November 2020, with poor response to medications. His condition progressed to akinetic-rigid state with bilateral foot dystonia in the following week. On the 9th of November, he was transferred to the emergency room for confusion. His oxygen saturation was subnormal (90%); however, he had no fever or any respiratory complaint. A nasopharyngeal swab reverse transcription-polymerase chain reaction (RT-PCR) for SARS-CoV-2 was conducted routinely for hospital admission, as per the Kuwait Ministry of Health (MOH) protocol. He tested positive for COVID-19 and was shifted to a specialized center. He developed fever and respiratory distress with low oxygen saturation, and he was intubated on the 10th day of illness. Computed tomography (CT) of the brain was normal. The patient was maintained on his regular treatment and was successfully extubated on the 19th of November. After discharge, he showed gradual improvement of consciousness and rigidity, and his feet dystonia improved; however, he did not reach his baseline prior to COVID-19 infection after 1 month.

## Discussion

We report a case of severe worsening of PD symptoms in the form of rigidity, akinesia, and foot dystonia, as the sole presenting features of COVID-19 infection. The patient was afebrile and lacked any of the cardinal features of the disease in the beginning. After a normal initial workup, the patient was found to be positive for COVID-19, which was incidentally requested. Other features of COVID-19 infection later ensued, with fever and respiratory symptoms. A gradual improvement occurred during the recovery phase without any specific therapy, apart from his usual anti-PD medications and treatment of COVID-19 infection as per MOH protocol.

Because of the unexplained, rapidly progressive worsening of PD symptoms, in a previously controlled PD patient, we believe that this presentation could be related to neuro-invasive potential of SARS-CoV-2, through sharing the same receptor angiotensin-converting enzyme 2 (ACE2), which can be found in the brain and mediate the disease process [3, 4].

The impact of COVID-19 on PD is largely speculative and coming mainly from case reports and case series. Generally, COVID-19 can affect PD through worsening of motor as well as non-motor symptoms, worsening of pre-existing dyspnea due to respiratory distress, as dyspnea may exist in up to 39% of PD patients, or through the development of new-onset parkinsonism [5].

Recently, four single-case reports have been published [6–9] describing the development of acute parkinsonism following COVID-19 infection. All four cases developed parkinsonian symptoms within 5–32 days of initial SARS-CoV-2 infection, and they showed evidence of reduced function of the nigrostriatal dopamine system, on brain imaging, similar to PD. Two of them [8, 9] developed these symptoms after only relatively mild COVID-19 infection, while the other 2 [6, 7] had moderate to severe infection requiring hospitalization, similar to our case.

Another community-based case control study of 12 PD patients in Italy [10] who developed COVID-19 suggested substantial worsening of motor and non-motor symptoms during mild to moderate COVID-19 illness, which is in line with our case; however, our case is unique in the aspect that worsening of PD was the sole initial presenting feature of COVID-19, in the absence of its common cardinal features. Furthermore, a 2021 study [11] of 10 PD cases from Wuhan, China, showed that worsened outcome was linked to older age, longer PD duration, and late stage PD.

Three potential mechanisms have been proposed for the rapid development of parkinsonism following COVID-19; first, a vascular insult from a hypercoagulable state associated with severe COVID-19 can directly damage the nigrostriatal system, akin to vascular parkinsonism. Second, systemic inflammation can trigger neuroinflammation and nigral dopamine neuron injury. Midbrain dopamine neurons express high levels of the ACE2 receptor and are believed to be vulnerable to SARS-CoV-2. Third, neurotropic features of SARS-CoV-2 can cause viral RNA to invade the brain. Neuropathological studies using immunostaining for aggregated α-synuclein have suggested that the PD starts in the olfactory system or in enteric nerves and then propagates to other brain regions [12–14].

Moreover, other additional factors in our case could be the increased levodopa requirement during acute illness, old age, being on advanced therapies, and having possible genetic PD that can make the patient vulnerable to immunologically mediated neuronal damage.

## Conclusion

To our knowledge, worsening of PD symptoms as the sole initial manifestation of SARS-CoV-2 infection, in the absence of other cardinal features of COVID-19 infection, has not been reported, expanding the disease clinical spectrum. In the era of COVID-19, we suggest that any unexplained worsening of PD symptoms, especially in advanced cases, should warrant for COVID-19 testing, even in the absence of its usual cardinal features.

## Limitations

Magnetic resonance imaging (MRI) could not be performed during COVID-19 infection because the patient was mechanically ventilated after admission.

## Data Availability

The data sets supporting the conclusion of this article are included within the article.

## Abbreviations

PD: Parkinson’s disease
COVID-19: Coronavirus disease 2019
SARS-CoV-2: Severe acute respiratory syndrome coronavirus 2
DA: Dopamine agonists
LCIG: Levodopa-carbidopa intestinal gel
H&Y: Hoehn and Yahr Scale
UPDRS: MDS-Unified Parkinson’s Disease Rating Scale
NMSS: Non-Motor Symptoms Scale
MoCA: Montreal Cognitive Assessment
RT-PCR: Reverse transcription-polymerase chain reaction
MOH: Ministry of Health
CT: Computed tomography
ACE2: Angiotensin-converting enzyme 2
MRI: Magnetic resonance imaging

## References

[1] Josephson SA, Kamel H (2020). Neurology and COVID-19. JAMA..

[2] Limphaibool N, Iwanowski P, Holstad MJV, Kobylarek D, Kozubski W. Infectious etiologies of parkinsonism: pathomechanisms and clinical implications. Front Neurol. 2019 Jun 19;10:652. doi: 10.3389/fneur.2019.00652. PMID: 31275235; PMCID: PMC6593078. 10.3389/fneur.2019.00652 PMC6593078 31275235

[3] Conde G, Pájaro LD, Marzola ID, Villegas YR, Salazar LR. Neurotropism of SARS-CoV 2: mechanisms and manifestations. Journal of the neurological sciences. 2020 Apr;8. 10.1016/j.jns.2020.116824 PMC7141641 32299010

[4] Hu J, Jolkkonen J, Zhao C. Neurotropism of SARS-CoV-2 and its neuropathological alterations: similarities with other coronaviruses. Neuroscience & Biobehavioral Reviews. 2020 Oct;19. 10.1016/j.neubiorev.2020.10.012 PMC7571477 33091416

[5] Sulzer, D., Antonini, A., Leta, V. et al. COVID-19 and possible links with Parkinson’s disease and parkinsonism: from bench to bedside. npj Parkinsons Dis. 6, 18 (2020). 10.1038/s41531-020-00123-0 10.1038/s41531-020-00123-0 PMC7441399 32885037

[6] Cohen ME, Eichel R, Steiner-Birmanns B, Janah A, Ioshpa M, Bar-Shalom R, Paul JJ, Gaber H, Skrahina V, Bornstein NM, Yahalom G (2020). A case of probable Parkinson’s disease after SARS-CoV-2 infection. The Lancet Neurology..

[7] Méndez-Guerrero, Antonio et al “Acute hypokinetic-rigid syndrome following SARS-CoV-2 infection.” Neurology 95.15 (2020): e2109-e2118. Web. 28 Dec. 2020. 10.1212/WNL.0000000000010282 32641525

[8] Faber I, Brandão PR, Menegatti F, de Carvalho Bispo DD, Maluf FB, Cardoso F (2020). Coronavirus disease 2019 and parkinsonism: a non-post-encephalitic case. Movement Disorders..

[9] Makhoul K, Jankovic J (2021). Parkinson’s disease after COVID-19. Journal of the Neurological Sciences..

[10] Cilia, R. et al. Effects of COVID-19 on Parkinson’s disease clinical features: a community-based case-control study. Mov. Disord. 10.1002/mds.28170 (2020), 2020. 10.1002/mds.28170 PMC7280741 32449528

[11] Zhai H, Lv Y, Xu Y, Wu Y, Zeng W, Wang T, Cao X, Xu Y. Characteristic of Parkinson’s disease with severe COVID-19: a study of 10 cases from Wuhan. Journal of Neural Transmission.:1-2. 10.1007/s00702-020-02283-y PMC7779096 33392827

[12] Brundin P, Nath A, Beckham JD (2020). Is COVID-19 a perfect storm for Parkinson’s disease?. Trends in neurosciences..

[13] Papa SM, Brundin P, Fung VS, Kang UJ, Burn DJ, Colosimo C, Chiang HL, Alcalay RN, Trenkwalder C (2020). MDS-Scientific Issues Committee. Impact of the COVID-19 pandemic on Parkinson’s disease and movement disorders. Mov Disord..

[14] Li WS, Chan LL, Chao YX, Tan EK (2020). Parkinson’s disease following COVID-19: causal link or chance occurrence?. J Transl Med.

